# Conjunctiva Necrosis Following Subconjunctival Amphotericin B Injection in Fungal Keratitis

**DOI:** 10.7759/cureus.5580

**Published:** 2019-09-05

**Authors:** Tham Han Shu, Adil Hussein, Mohd Razali Kursiah

**Affiliations:** 1 Ophthalmology, School of Medical Sciences, Universiti Sains Malaysia, Kubang Kerian, MYS; 2 Ophthalmology, Hospital Raja Permaisuri Bainun, Ipoh, MYS

**Keywords:** conjunctiva necrosis, subconjunctival amphotericin b injection, fungal keratitis

## Abstract

A 30-year-old Bangladeshi gentleman presented with history of sand entering his left eye and was diagnosed as having fungal keratitis by private ophthalmologist. He was treated with three doses of conventional subconjunctival amphotericin B injections (1.5 mg of amphotericin B and 1.2 mg of deoxycholate) over the inferior bulbar conjunctiva and topical antibiotics. Subsequently, he developed conjunctival necrosis over the site of injections and there was no clinical improvement of the keratitis. He was then treated with intensive antifungal and antibiotics eye drops. Debridement of epithelial plug was done and he was given intracameral amphotericin B injection. There was gradual improvement observed then with conjunctival epithelialization. The conjunctival tissue was completely healed after three months along with the corneal ulcer.

Subconjunctival injection of Amphotericin B (AMB) may be considered as an adjunct therapy in severe fungal keratitis to address the issue of compliance. Close monitoring is needed due to its known complication of scleritis, scleral thinning and conjunctival necrosis. Liposomal AMB which is known to cause less toxicity given via subconjunctival injection in human subjects needs to be further studied.

## Introduction

Fungal keratitis is a serious sight-threatening inflammation of the cornea which can cause formation of hypopyon, ulcer, suppuration, destruction and even perforation of the corneal tissues [1]. There appears to be a strong geographical influence on different types of causative fungal infection. Tropical climates show a higher percentage of filamentous fungi infections whereas temperate climates show a preponderance of yeast infections [2]. Trauma appears to be the main predisposing factor, occurring in 40-60% of patients [3, 4]. Other risk factors include previous ocular surgery, ocular surface disease, chronic use of corticosteroid (topical or systemic form) and contact lens wear [5-7]. Filamentous fungal keratitis usually occurs in healthy young males involved in agricultural or outdoor work following corneal trauma by vegetative material [8]. These fungi do not penetrate intact corneal epithelium, invasion is secondary to trauma [9-11]. Management of fungal keratitis largely depends on the suspected causative fungal, the severity of keratitis and the route of administration. Antifungal can be given such as topical eye drops, subconjunctival injection, intrastromal, intracameral or intravitreal injection.

Amphotericin B has a broad spectrum of antifungal activity and remains a potent antifungal agent advocated for use both systematically and locally in the treatment for ocular mycosis [12, 13]. Here, we report a case of fungal keratitis treated with conventional subconjunctival amphotericin B and subsequently developed conjunctival necrosis.

## Case presentation

**Figure 1:**
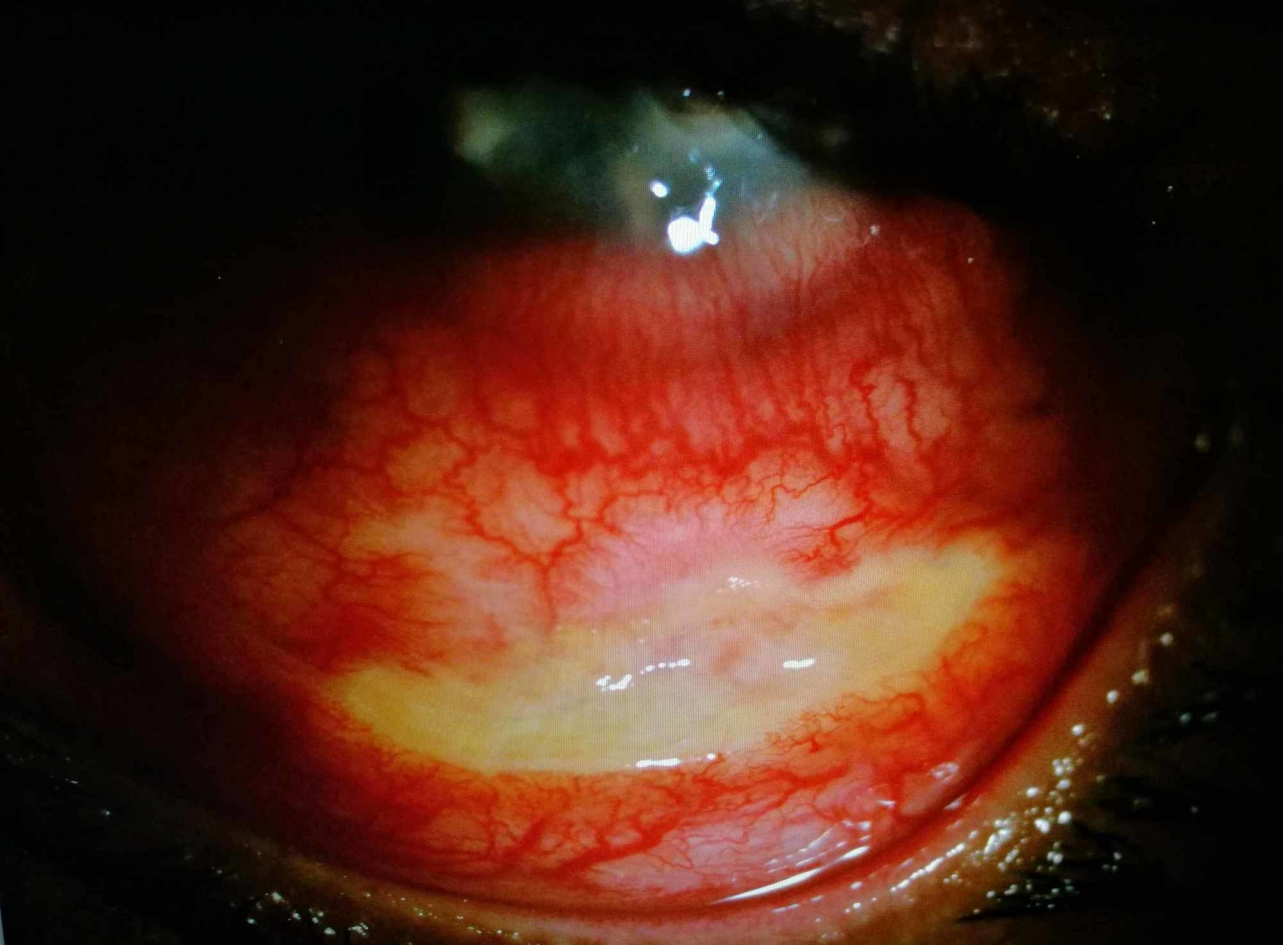
Slit lamp examination showed necrosis of inferior bulbar conjunctiva which corresponds to the site of injections. There was no scleral inflammation.

**Figure 2:**
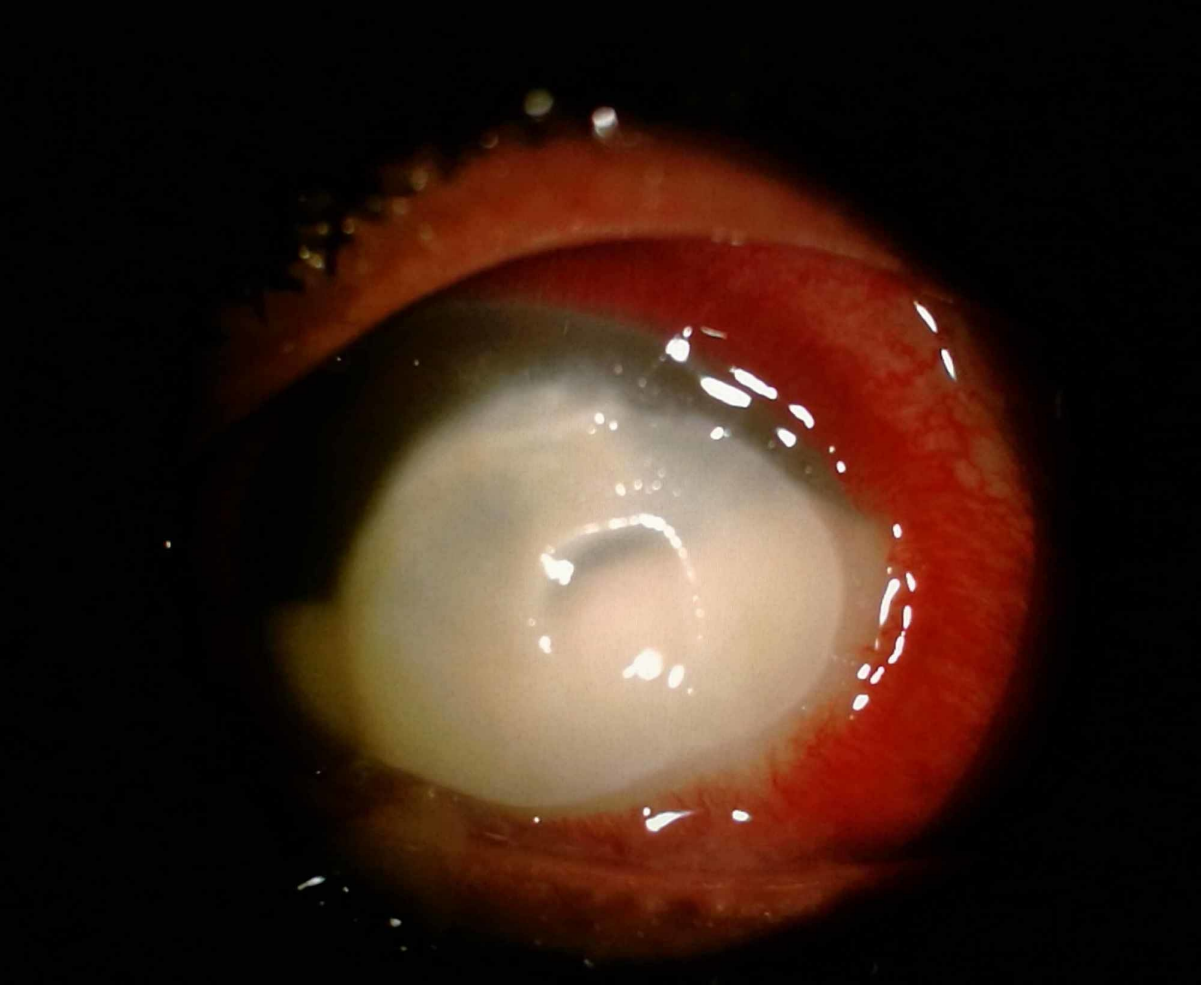
Slit lamp examination showed a dense central stromal infiltrate with central thinning and dense epithelial plug.

A 30-year-old Bangladeshi gentleman presented with history of sand entering his left eye and was diagnosed as having fungal keratitis by private ophthalmologist. He was treated with topical antibiotics and three doses of conventional subconjunctival amphotericin B injections (1.5 mg of amphotericin B and 1.2 mg of deoxycholate) over the inferior bulbar conjunctiva (Figure 1). Due to financial constraint, he was referred to government hospital for further management. The visual acuity of his left eye upon review was counting finger. The left eye conjunctiva was injected with yellowish discharge. There was a dense central corneal stromal infiltrate measuring 5 mm x 7 mm with central thinning (Figure 2). The edges were feathery with presence of satellite lesions. There was thick epithelial plaque surrounding an area of thinning of center cornea. No obvious hypopyon level was seen. Linear conjunctiva necrosis measuring 4 mm noted at the inferior fornix corresponded to the site of subconjunctival amphotericin B injection. The base showed grey to white scleral layer with no evidence of subconjunctival nodules or scleral inflammation. The surrounding conjunctiva was mildly congested. Corneal scrapping was not performed in view of partially treated corneal ulcer. He was treated with intensive antifungal and antibiotics eye drops. Debridement of epithelial plug was done and he was given intracameral amphotericin B injection subsequently during hospitalization. There was gradual improvement observed over three weeks where the base of the conjunctival necrosis appeared clean with evidence of scleral vascularization. Conjunctival epithelialization occurred from the edge of the surrounding healthy conjunctiva tissue. The conjunctival tissue was completely healed after three months along with the corneal ulcer.

## Discussion

Amphotericin B (AMB) was the first broad-spectrum antifungal agent to be discovered. It belongs to the family of polyene macrolide antibiotics [14]. It has broad spectrum of activity against yeast and molds whereby it binds to ergosterol in the fungal cell membrane causing ion leakage and cell death. The initial formulation available was amphotericin B deoxycholate (DAmB) which was developed in the 1950s. A newer lipid formulation of amphotericin B known as the liposomal amphotericin B was developed for nearly 20 years to treat a broad spectrum of fungal infections.

In this case, the patient was given regular topical AMB. To further enhance intraocular penetration of AMB, an additional of three doses of subconjunctival AMB (1.5 mg of amphotericin B and 1.2 mg of deoxycholate) was given by the private ophthalmologist. Subconjunctival route was an option in this case because the patient was a foreigner with financial constraint and low adherence to the treatment was anticipated. Treatment with subconjunctival injection of AMB was debatable due to reports of conjunctival necrosis, scleritis and scleral thinning [15, 16]. In 1973, Randall et al. reported a case of fungal ulcer developing subconjunctival nodule following subconjunctival AMB injection. Microscopic examination revealed a fibrosing histiocytic nodule [17]. Retrospective study done by Nada and Bor’i between 2010 to 2015 reported different treatment modalities of fungal keratitis by antifungal agents which included a combination of topical natamycin and subconjunctival amphotericin B in one of the treatment groups. This group was noted to achieve high curative rate just second to the group treated with a combination of intrastromal injection of AMB and topical fluconazole eye drops. There was no complication reported from subconjunctival AMB injection in this study [18]. Similarly, case series reported by Carrasco and Genesoni reviewed four cases of severe fungal keratitis treated with two to eight injections of subconjunctival conventional AMB as an adjunct to topical fluconazole eye drops [19]. All patients reported burning and pain during the injection which resolved spontaneously shortly after injection. The only changes noted in the conjunctiva were chemosis, small hemorrhages at the site of injection and yellow appearance of the conjunctiva following injection. None of the cases develop subconjunctival nodule, ulceration or necrosis as reported by previous studies.

In this patient, he developed conjunctival necrosis exposing the underlying grey to white scleral layer following three doses of subconjunctival AMB injection, with no improvement of the fungal ulcer. This might be due to the poor ocular penetration of topical antifungal eye drops which was hindered by the dense epithelial plug. Periodic debridement of the corneal epithelial plug was done in conjunction with intensive hourly topical antifungal subsequently and it leads to drastic clinical improvement. Animal studies revealed in rabbits whose corneal epithelium had been removed, therapeutic levels were reached in the corneal stroma [12-14]. It was shown that the large molecule’s size of AMB hinders penetration into cornea when the epithelium is intact.

AMB deoxycholate has been the only antifungal agent for the treatment of invasive fungal disease for many decades. However, due to its dose-limiting toxicity, new less toxic formulation, which is the liposomal AMB, has been developed. This new lipid formulation of AMB retained its antifungal activity by incorporating into a liposome bilayer, hence its toxicity is significantly reduced [20]. However, there was limited data on the toxicity and efficacy of topical application of the liposomal formulation of AMB. Kaji et al. evaluated the efficacy and toxicity of these two formulations in an animal study of white rabbits [13]. The animals were divided into three groups receiving either subconjunctival injection of deoxycholate AMB, liposomal AMB or deoxycholate. The result showed that deoxycholate AMB and deoxycholate gave almost the same degree of ocular toxicity evidenced by severe corneal oedema, corneal epithelial erosion, corneal stromal oedema with inflammation and corneal endothelial cell loss. In contrast, liposomal AMB was shown to have reduced toxicities.

## Conclusions

Subconjunctival injection of AMB may be considered as an adjunct therapy in severe fungal keratitis to address the issue of compliance. However, close monitoring is needed due to the reported complications of conjunctival necrosis, scleritis and scleral thinning. Liposomal formulation of AMB was known to have reduced toxicity compared to the conventional formulation, however, its efficacy and toxicity in subconjunctival injection in humans should be further studied.
